# Navigating antiphospholipid syndrome: from personalized therapies to cutting-edge research

**DOI:** 10.1093/rap/rkaf005

**Published:** 2025-01-08

**Authors:** Karen Kortright-Maldonado, Bruno Eduardo Reyes-Torres, Lilian Stephany Cabrera-Lopez, Pedro Rodríguez-Henríquez, Erika Karina Tenorio-Aguirre, Froylan D Martínez-Sánchez

**Affiliations:** Department of Internal Medicine, Hospital General “Dr. Manuel Gea González”, Ciudad de Mexico, Mexico; Facultad de Medicina, Universidad Nacional Autonoma de Mexico, Ciudad de Mexico, Mexico; Department of Internal Medicine, Hospital General “Dr. Manuel Gea González”, Ciudad de Mexico, Mexico; Department of Internal Medicine, Hospital San Angel Inn Universidad, Ciudad de Mexico, Mexico; Department of Rheumatology, Hospital General “Dr. Manuel Gea Gonzalez”, Ciudad de Mexico, Mexico; Department of Internal Medicine, Hospital General “Dr. Manuel Gea González”, Ciudad de Mexico, Mexico; Department of Internal Medicine, Hospital General “Dr. Manuel Gea González”, Ciudad de Mexico, Mexico; Facultad de Medicina, Universidad Nacional Autonoma de Mexico, Ciudad de Mexico, Mexico

**Keywords:** APS, aPLs, artificial intelligence, gut microbiome

## Abstract

APS is an autoimmune disorder characterized by thrombosis and pregnancy complications, primarily driven by aPLs such as LA, aCL and anti-β2 glycoprotein I (a-β2GPI). Despite advances in anticoagulation therapies, managing refractory APS cases remains challenging. Emerging therapies, including rituximab, eculizumab and HCQ, show potential in addressing the underlying mechanisms of APS. Additionally, research into genetic and environmental factors, particularly the gut microbiome’s role through molecular mimicry, suggests new therapeutic pathways. Diagnostic advancements, such as the adjusted Global Antiphospholipid Syndrome Score (aGAPSS), metabolomic profiling and MRI, have improved risk stratification and early detection. Non-traditional biomarkers like anti-phosphatidylserine/prothrombin (aPS/PT) and anti-Domain I antibodies further enhance risk assessment. Future research should aim to validate these approaches, optimizing patient outcomes and minimizing long-term APS complications.

Key messages

- Early diagnosis and personalized treatment approaches are crucial to managing APS and preventing complications.
- Advanced diagnostic tools like biomarkers and MRI improve risk assessment for APS patients.
- New therapies targeting the immune system offer hope for patients with refractory APS.

## Introduction

APS is a systemic autoimmune disorder characterized by thrombosis in arterial, venous or microvascular vessels and pregnancy complications, occurring in the presence of persistent aPLs such as LA, anti-β2 glycoprotein I (a-β2GPI) and aCL [1, 2]. These antibodies play a central role in APS pathogenesis [1, 3]. APS is clinically significant due to its potential for life-threatening thrombotic complications, including deep vein thrombosis, stroke and myocardial infarction, even in younger populations [3]. The severity of these outcomes underscores the importance of early diagnosis and a multidisciplinary treatment approach to mitigate risks [1, 2]. By affecting multiple organ systems, APS increases the risk of morbidity and mortality, placing a significant burden on patients and emphasizing the need for timely and accurate diagnosis [3]. This review provides an overview of APS, covering its pathogenesis, clinical manifestations and diagnostic and treatment challenges.

### Epidemiology

The annual incidence of APS is approximately 2.1 per 100 000, with a prevalence of 50 per 100 000 in the general population [3]. APS can present as a primary condition or secondary to other diseases, most notably SLE, where its prevalence ranges from 8.9% to 27% among SLE patients [4, 5]. Approximately 8% of patients with primary APS may eventually develop SLE during long-term follow-up [6, 7]. Accurately estimating APS frequency is difficult due to changing classification criteria, lack of standardized tests for aPL detection and challenges in confirming antibody positivity over 12 weeks [8]. Larger, population-based studies are needed, particularly in diverse racial and ethnic groups, to clarify APS incidence and prevalence [7, 8]. As classification criteria evolve, previously undiagnosed or misclassified patients may be redefined as having APS, potentially changing current epidemiological estimates [1, 8].

### Pathophysiology

The pathophysiology of APS is complex, involving the interaction of aPL with various components of the immune and coagulation systems [1, 2]. APS is primarily associated with antibodies targeting β2GPI, which can bind to endothelial cells and trigger a pro-thrombotic response [9, 10]. These aPL activate endothelial cells, monocytes, platelets and neutrophils, contributing to the development of thrombosis through tissue factor expression, complement activation and neutrophil extracellular traps (NETs) [9, 10].

**Figure 1.**
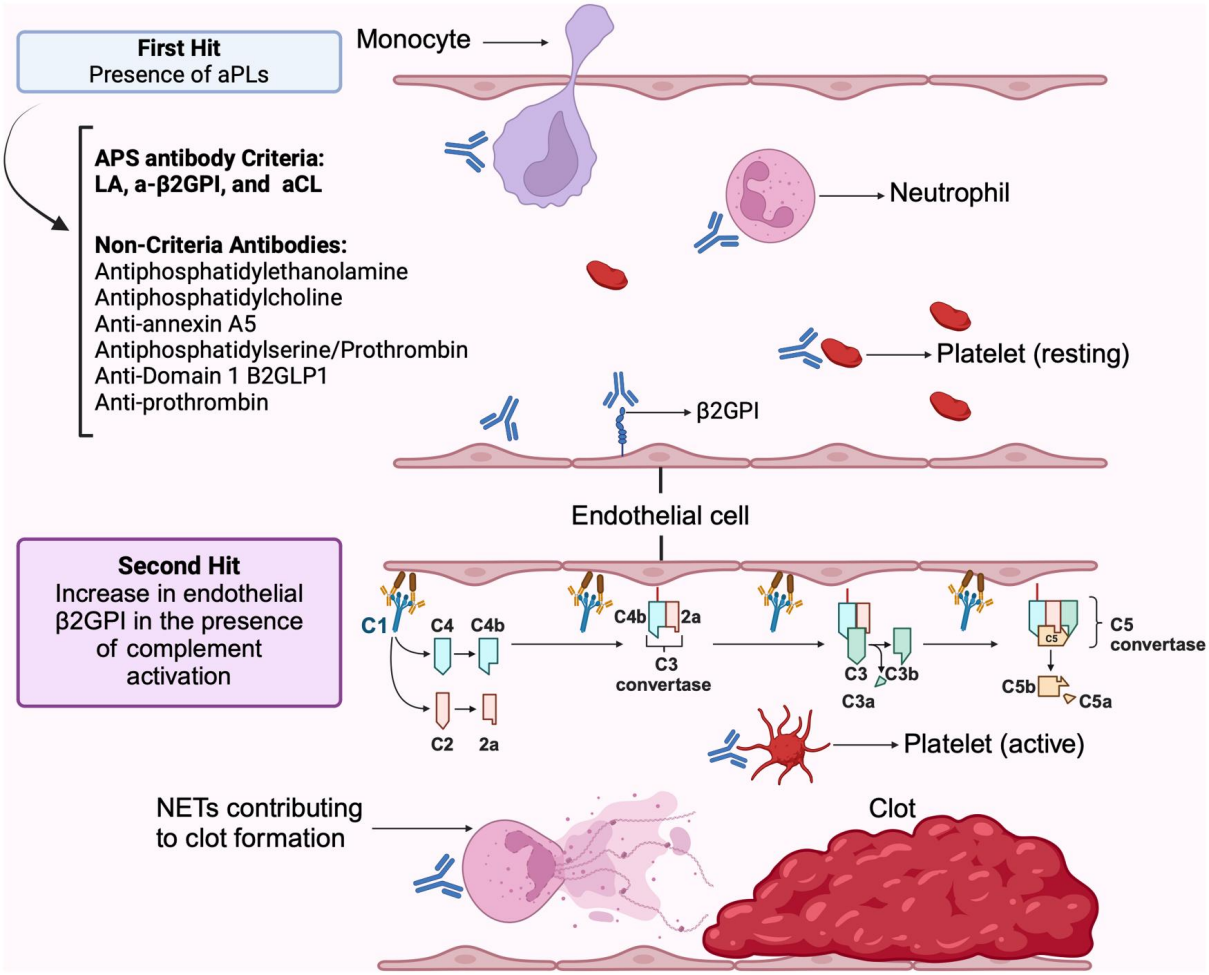
Pathophysiology of APS through the two-hit hypothesis. Clot formation in APS begins with the first hit, where aPLs such as LA, anti-β2 glycoprotein I (a-β2GPI) and aCL bind to β2GPI on endothelial cells, platelets, neutrophils and monocytes. Non-criteria antibodies (e.g. antiphosphatidylethanolamine, antiphosphatidylserine/prothrombin and others) have been suggested to contribute to the pro-thrombotic state. The second hit involves complement activation, leading to endothelial cell activation and tissue damage. The complement cascade activates C3 and C5 convertase, generating C5a and the membrane attack complex. Neutrophils release neutrophil extracellular traps (NETs), which promote clot formation. Activated platelets and endothelial cells amplify the thrombotic response, resulting in thrombosis

Complement activation plays a central role in APS, particularly in mediating the tissue damage seen in both vascular and obstetric manifestations. In animal models, complement inhibition has been shown to reduce aPL-mediated effects, supporting its role in disease pathogenesis [2, 9]. The ‘two-hit hypothesis’ suggests that while persistent aPLs create a pro-coagulant environment (the first hit), a second trigger, such as infection or inflammation, is required to initiate clot formation (Fig. 1) [9].

In APS nephropathy, which occurs in primary APS and SLE-associated APS, microvascular thrombosis leads to renal damage, resulting in thrombotic microangiopathy and chronic vascular lesions [10]. Despite advancements in understanding the histopathological features of APS nephropathy, there remain challenges in identifying patients at higher risk and defining targeted therapeutic strategies [1, 10].

### Treatment cornerstones

Anticoagulation therapy is a cornerstone of APS management [2]. However, current prevention and treatment strategies are unsuccessful in approximately 20–30% of obstetric APS cases and more than 30% of thrombotic APS cases [9, 11]. This highlights the need for better treatment approaches, as current stratification tools are insufficient to identify which patients would benefit most from antithrombotic prophylaxis and optimal treatment intensity [9]. While anticoagulation remains the primary treatment, it is not practical for all APS patients, especially those with refractory conditions like catastrophic APS (CAPS) [11]. Introducing biologics, such as rituximab and eculizumab, offers a promising alternative for managing these cases [11, 12]. Additionally, new oral drugs that block complement activation are showing promise. Still, their safety profiles and potential side effects, such as triggering harmful secondary events in aPL-positive patients, remain under evaluation [9].

## Personalized medicine in APS

### Genetic and environmental factors influencing APS

Genetic and environmental factors significantly influence the pathogenesis of APS. One important genetic factor is the methylenetetrahydrofolate reductase (MTHFR) C677T mutation, which has been identified as a risk factor for arterial thrombosis, particularly in the Chinese Han population [13]. Moreover, other thrombophilic gene polymorphisms, such as PAI-1 4G/5G, factor V Leiden G1691A, prothrombin G20210A and antithrombin III deficiency, have been implicated in APS, especially for increasing the risk of venous thrombosis and recurrent pregnancy loss (RPL) [13–15]. Fibrinogen gene polymorphisms, such as FGA G1233A and FGB A9692G, have also been studied, though their contributions to thrombosis risk remain under investigation [13].

Environmental factors also play a crucial role, particularly in the gut microbiome [14]. An altered gut microbiome composition has been observed in APS patients, with specific microbes like *Roseburia intestinalis* potentially triggering immune responses through molecular mimicry by resembling APS autoantigens, which may lead to increased disease activity and severity [14]. Infections, such as those caused by *Mycoplasma pneumoniae*, HIV and hepatitis C, are known to induce aPL production and may trigger APS in genetically predisposed individuals [15]. These microorganisms affect APS by influencing immune responses and non-immunological mechanisms, such as alterations in diet and microbial short-chain fatty acid production [14, 15].

### Individualized risk stratification for thrombotic events

Effective risk stratification in APS involves assessing factors contributing to thrombotic events, including triple positivity for aPL, the adjusted Global Antiphospholipid Syndrome Score (aGAPSS), thrombocytopenia, metabolomic profiles and arterial stiffness. Additional tools such as cluster analysis and the Damage Index for APS (DIAPS) provide further insights into risk assessment [16–21].

#### aGAPSS score

The aGAPSS is a valuable tool for predicting thrombotic events, especially in patients with primary obstetric APS [19]. It incorporates cardiovascular risk factors and aPL profiles to stratify patients for long-term thromboprophylaxis. Notably, patients with an aGAPSS score ≥7 have been found to benefit from low-dose aspirin for thromboprophylaxis [20].

#### Metabolomic profiles

Recent studies have identified distinct metabolomic signatures correlating with thrombotic risk in patients with APS. High-risk profiles are characterized by elevated levels of atherogenic lipoproteins, including very-low-density lipoprotein and specific high-density lipoproprotein subsets, alongside altered concentrations of fatty acids, amino acids and inflammatory metabolites [22]. These metabolic changes are associated with increased cardiovascular risk and reflect heightened oxidative stress and inflammation. Additionally, disruptions in glucose and creatinine levels have been observed, further linking metabolomic profiles to vascular dysfunction in patients with APS. Identifying these profiles offers the potential for stratifying patients based on their thrombotic risk and tailoring more targeted therapeutic approaches [22].

#### Arterial stiffness

Chronic inflammation in APS can lead to endothelial dysfunction and arterial stiffness, predictors of cardiovascular events. Measuring arterial stiffness through techniques like pulse wave velocity and the augmentation index (AIx) helps assess cardiovascular risk. APS patients have been found to exhibit increased AIx, indicating a higher burden of cardiovascular disease [23, 24].

#### Damage index for APS

The DIAPS was developed to quantify cumulative organ damage in APS patients, particularly those with thrombotic APS [16]. This 38-item index has demonstrated strong validity and is associated with poorer quality of life. An increase in DIAPS within the first 5 years of illness predicts higher mortality [16, 25]. However, the DIAPS has certain limitations, including inconsistencies in scoring, particularly regarding the inclusion of sequelae, the absence of weighted scoring for individual items and limited validation across diverse populations. Additionally, the index may benefit from incorporating more clinical manifestations specific to APS to improve its comprehensiveness and applicability [25].

### Tailoring treatment based on patient profiles

Tailoring treatment in APS involves a multimodal approach, considering cardiovascular risk assessment, metabolomic profiling, and identifying high-risk patients through tools like cluster analysis and DIAPS. Vitamin K antagonists (VKAs) remain the foundation for thrombosis management, and combining antiplatelet agents with warfarin has shown potential for reducing recurrence [16, 19].

#### High-risk profiles

Managing cardiovascular risk in APS requires evaluating disease-specific factors such as thrombocytopenia, arterial stiffness and cardiometabolic traits often seen in high-risk conditions like diabetes [26, 27]. The EULAR guidelines recommend following general population standards for managing hypertension and hyperlipidaemia, using tools like the SCORE system to guide treatment decisions [26–28]. The SCORE system evaluates traditional cardiovascular risk factors, including gender, age, tobacco use, blood pressure and total cholesterol, to guide treatment decisions in APS patients [28]. Incorporating APS-specific factors like aPL profiles and thrombotic history refines this assessment. Moreover, patients with triple positivity are at the highest risk for thrombotic events and relapses [19]. These patients often experience more frequent arterial and non-criteria events. Long-term anticoagulation with VKAs is strongly recommended for high-risk profiles, while direct oral anticoagulants (DOACs) remain controversial [29].

High-risk pregnancies in patients with APS, particularly those unresponsive to standard heparin and low-dose aspirin treatments, require additional interventions [26, 27]. Therapies like HCQ, IVIG, plasma exchange and low-dose prednisolone have been shown to improve live birth rates in certain high-risk APS pregnancies [30–33]. While these approaches are promising, the optimal strategy for managing these pregnancies remains debated [31, 33].

#### Low-risk profiles

Asymptomatic carriers of aPL generally present a low risk for thrombotic events. These patients tend to have lower Factor XI levels and fewer clinical manifestations, correlating with a reduced risk of significant complications [19, 26]. Patients with single aPL positivity are less likely to experience thrombotic events and often fall into a lower-risk category [1, 26]. In such cases, less aggressive anticoagulation may suffice, and treatments like low-dose aspirin or DOACs may be viable options, though further studies are required to confirm their safety [19, 32]. The heterogeneity in APS patients underscores the need for personalized treatment plans to optimize outcomes and reduce complications [26, 32–34].

## Emerging therapies: a comparison with traditional treatments

### Vitamin K antagonists vs. direct oral anticoagulants

Several studies have compared the efficacy of DOACs and VKAs in preventing thromboembolic events in APS patients. A meta-analysis of randomized clinical trials found that DOACs, particularly rivaroxaban, had a higher rate of thromboembolic recurrences than VKAs, especially in patients with arterial index events or triple aPL positivity [35–38]. DOACs significantly increased the risk of arterial thrombosis, including stroke, compared with VKAs [36, 37]. Subgroup analyses confirmed higher risks for patients with triple positivity or a history of arterial thrombosis treated with DOACs [37].

Although the risk of major bleeding did not differ significantly between DOACs and VKAs, the elevated risk of arterial thrombosis with DOACs raises safety concerns, particularly for high-risk APS patients [36, 38]. Clinical guidelines recommend avoiding DOACs in patients with a history of arterial thrombosis or triple aPL positivity, favouring VKAs as the first-line treatment for high-risk individuals. DOACs may be considered for low-risk patients without a history of arterial events [26, 27, 37, 38].

### Role of rituximab, eculizumab and HCQ

While anticoagulation remains the primary treatment for APS, there is growing interest in targeted therapies like rituximab, eculizumab and HCQ, especially in cases where conventional anticoagulation proves inadequate. These therapies aim to address the underlying immunological mechanisms of APS [26, 39].

#### Rituximab

Rituximab, a monoclonal antibody targeting CD20, has shown potential in treating refractory APS cases. Numerous studies and case reports have documented rituximab’s effectiveness in managing complex APS manifestations, including recurrent thrombosis, neurological complications and CAPS [39–41].

For example, a case report highlighted the successful use of rituximab in a patient with APS-associated crescendo stroke events after standard anticoagulation therapies had failed [41]. Similarly, a retrospective case series from Israel demonstrated favourable outcomes in 80% of APS patients treated with rituximab, particularly when a 4-week protocol was followed [42]. Response to rituximab treatment was associated with a rituximab protocol of 375 mg/m^2^ administered weekly for 4 weeks, compared with a fixed dose of 1000 mg administered twice (100% vs. 65%; *P* = 0.01). Complete response was associated with a decrease in aPL titres within 4–6 months post-treatment, whereas no significant change in aPL titres was observed in patients with partial or no response [42].

#### Eculizumab

Eculizumab, a C5 complement inhibitor, has emerged as a promising treatment for APS, particularly in patients with refractory CAPS, where conventional therapies often fail to lower the high mortality rates associated with the condition. The complement system plays a crucial role in APS pathogenesis, and inhibiting its activation has been shown to reduce the thrombotic and inflammatory processes driving the disease [43, 44].

The 14th International Congress on Antiphospholipid Antibodies emphasized the need for controlled clinical trials to assess the efficacy of complement inhibitors like eculizumab in APS management [44]. This drug has been particularly beneficial in CAPS, but its use in non-catastrophic cases still requires further research.

#### Hydroxychloroquine

HCQ, a drug initially developed for malaria, has demonstrated significant immunomodulatory and antithrombotic effects, making it a valuable agent in managing APS [43, 45]. Bertolaccini *et al.* showed that HCQ can inhibit complement activation, a key APS mechanism that reduces tissue factor expression and attenuates inflammatory pathways [39, 45]. This action is crucial in preventing thrombotic events and pregnancy-related complications in APS, including foetal loss and placental abnormalities [40]. Moreover, the drug reduces aPL titres and improves endothelial function, enhancing its protective effect in thrombotic and obstetric APS [39, 45].

Kravvariti *et al.* highlighted HCQ’s role in preventing thrombosis in APS patients, demonstrating its significant ability to reduce thrombotic events when combined with standard anticoagulation therapy. Their study also showed that HCQ might reduce vascular inflammation by modulating Toll-like receptor pathways and inhibiting platelet aggregation [46]. In addition to its antithrombotic properties, HCQ has been suggested for use in refractory APS and CAPS cases and in combination with other therapies, such as low-dose aspirin and heparin, to improve pregnancy outcomes [39, 40]. These findings underline the importance of HCQ as a cornerstone of APS management, particularly in high-risk populations where standard anticoagulation alone may not suffice [19, 27].

### Clinical studies outcomes: successes and challenges

Assessing aPL profiles, especially LA positivity and ‘triple aPL positivity’, is crucial for risk stratification, helping to identify patients at the highest risk of thrombotic events. Developing a panel of criteria and non-criteria aPL tests will further improve risk stratification [18, 19, 47]. Regarding novel therapeutic approaches, targeting the complement and the mammalian target of rapamycin (mTOR) pathways and B cell inhibition offers promising strategies for difficult-to-treat patients with APS [47–51]. Additionally, the discovery of pathogenic features like domain I-specific a-β2GPI antibodies has highlighted endothelial and complement activation as crucial drivers of APS, paving the way for innovative pharmacological tools such as novel anticoagulants and complement inhibitors like eculizumab [49, 51, 52]. Unfortunately, there is scarce data on evaluating emerging therapies for APS.

As mentioned above, rituximab has been particularly effective in cases of refractory APS, reducing aPL titres and improving outcomes in about 80% of patients, especially in CAPS and those with recurrent thrombosis [39–41, 50]. However, large-scale studies are still required to further establish its efficacy and safety.

HCQ has been beneficial in obstetric APS, improving pregnancy outcomes when combined with anticoagulants [52]. Statins, particularly fluvastatin, have shown promise in reducing pro-inflammatory and pro-thrombotic markers, justifying their use as adjunctive treatments in refractory APS cases [48, 51]. Statins are hypothesized to be effective in APS by inhibiting the mTOR pathway [48]. In APS, overactivation of mTOR may lead to endothelial dysfunction and increased thrombosis risk. By inhibiting this pathway, statins may reduce inflammation and vascular dysfunction, potentially lowering the risk of thrombotic events [48, 51, 52]. While these therapies show significant promise, their success depends on overcoming the challenges of treatment resistance, recurrence and long-term safety, underscoring the need for continued research in APS management.

## Advances in diagnostics: multimodal imaging and molecular markers

### Role of MRI, PET and other imaging techniques

MRI is useful for detecting brain and heart abnormalities in APS patients, especially those at high risk of cardiovascular or cerebrovascular events [53, 54]. Brain MRI frequently reveals infarcts, white matter hyperintensities (WMH) and brain atrophy in APS [53]. One study showed that 36% of APS patients had WMH, compared with 12% of controls, with a higher prevalence in patients exhibiting neurological symptoms [54]. Moreover, periventricular hyperintensities were seen in 44% of APS patients, particularly those with cognitive dysfunction or seizures [54].

Cardiac MRI has proven invaluable in detecting silent myocardial ischaemia and fibrosis, even in asymptomatic APS patients [55]. A 2019 case–control study reported that 36.3% of APS patients had late gadolinium enhancement, indicating myocardial fibrosis, compared with 0% in healthy controls (*P* < 0.001). Furthermore, the myocardial perfusion reserve index (MPRI) was significantly lower in APS patients, with an average of 1.5 compared with 2.7 in controls (*P* < 0.001) [55]. These findings suggest that APS patients are at risk of silent cardiac damage, independent of traditional cardiovascular risk factors, and underscore the utility of stress cardiac MRI in early detection. While conventional MRI techniques are helpful, advanced methods like magnetization transfer imaging can detect subclinical central nervous system lesions not visible on standard MRI. These techniques may help identify subtle changes that precede more overt neurological manifestations, although further studies are needed to confirm their routine clinical use [56].

### The use of biomarkers for early detection

In APS, biomarkers are crucial in early detection and risk stratification for thrombotic and obstetric events. While traditional aPLs remain central to diagnosis, several emerging biomarkers offer enhanced diagnostic precision and predictive power [57, 58].

1. **Non-traditional antibodies**: Anti-phosphatidylserine/prothrombin antibodies (aPS/PT) have been shown to significantly correlate with LA positivity, thrombosis risk and classification of APS patients. These antibodies offer additional stratification potential, especially in high-risk patients, and have been highlighted as valuable in risk assessment for vascular events and obstetric complications [58, 59].
2. **Anti-Domain I antibodies (anti-DI)**: Anti-DI antibodies target Domain I of β2GPI and are particularly associated with high-risk APS patients, correlating more strongly with clinical manifestations than traditional assays. Studies have shown that anti-DI positivity provides a higher odds ratio for thrombotic and obstetric events than other aPL tests, especially when combined with conventional aPL positivity [60, 61].
3. **Post-translational modifications of aPL**: Glycosylation patterns of aPL antibodies, including sialylation, fucosylation and galactosylation, modulate their inflammatory and pro-thrombotic potential. For example, decreased sialylation of IgG has been linked to enhanced thrombosis risk. Quantifying these post-translational modifications can offer additional prognostic insights into thrombosis risk in APS patients [61].
4. **Neutrophil extracellular traps**: NETs have emerged as critical players in APS pathophysiology, promoting thrombosis by providing scaffolds for platelet adhesion and coagulation. Elevated levels of NETs and anti-NET antibodies in the serum of APS patients have been linked to disease severity, making them promising biomarkers for risk assessment [22].
5. **Oxidative stress biomarkers**: Paraoxonase 1 (PON1) activity has shown potential for thrombotic risk in APS due to its modulating oxidative stress and protecting against lipid peroxidation. Reduced PON1 activity has been associated with increased thrombotic events [61].
6. **MicroRNAs (miRNAs)**: Specific miRNA signatures have been identified in APS patients, with implications for gene regulation linked to thrombosis and atherosclerosis. Circulating miRNAs, such as miR-19b and miR-20a, show promise as biomarkers for APS severity and progression, adding another layer to personalized risk stratification [22].

### Improving diagnostic accuracy through laboratory testing

Combining laboratory tests enhances diagnostic accuracy for APS [62]. Sciascia *et al.* evaluated 23 combinations of aPL. They found that the combination of LA, aβ2GPI and aPS/PT antibodies produced the highest diagnostic accuracy for thrombosis and pregnancy loss, achieving a specificity of over 90% [62]. Accessing multiple aPLs through multiplex assays further improves diagnostic precision. For instance, Sénant *et al.* demonstrated that multiplex testing for aCL, anti-β2GPI and factor II enhances diagnostic performance in primary APS, resulting in a 15% increase in sensitivity compared with traditional ELISA. Multiplex assays also enable the detection of non-traditional aPL antibodies, expanding their diagnostic utility [63].

#### APS and pregnancy: novel management strategies

Obstetric complications in APS patients pose significant risks to both maternal and foetal health. Likewise, aPLs are strongly linked to pregnancy-related issues, with miscarriage rates reaching up to 50%, elevated risks for preeclampsia (15–20%) and intrauterine growth restriction (10–30%) [9, 18–20]. Despite using antithrombotic therapies like low-dose aspirin and heparin, 30% of pregnancies still experience complications such as preeclampsia, placental abruption and foetal growth restriction, collectively referred to as late placental complications, highlighting the need for new treatments [19].

Children born to aPL-positive mothers may have a higher risk of neurocognitive issues, although more research is needed [19, 20]. Women with obstetric APS have a 10–15% risk of postpartum venous and arterial thrombosis and may develop psychiatric or mood disorders later in life [20]. Early identification of risk factors through clinical markers and biomarkers, such as aPS/PT antibodies and LA, combined with imaging techniques like MRI, improves the prediction of complications. Multiplex assays, which assess multiple aPLs, offer 15–20% better sensitivity for risk stratification than traditional tests [59].

Recent advances in managing RPL focus on both immunological and genetic aspects, which offer new strategies for improving outcomes in affected patients [64]:

1. **Endometrial immunological profile**: A study found that tailoring treatments based on the endometrial immunological profile, obtained from the material of an endometrial biopsy, increased the live birth rate by approximately 50% in women with RPL [64].
2. **Immunological interventions**: Immunomodulatory therapies like IVIg have shown variable success in restoring immunological balance. In some cases, IVIg has improved pregnancy outcomes in APS patients, though success rates vary depending on the patient profile.
3. **Chromosomal analysis**: The introduction of chromosomal microarray analysis has enhanced the identification of genetic causes of RPL, reducing the percentage of unexplained cases from more than 50% to less than 10% [65]. This reduction provides a more comprehensive explanation for the losses, enabling more focused and personalized management strategies.
4. **Immunological therapies**: It has been suggested that steroids and other immunomodulatory therapies might significantly improve obstetric outcomes, with some studies showing an improvement in live birth rates by up to 35% in women with otherwise unexplained RPL [65].

#### Microbiome and environmental triggers in APS

**Figure 2.**
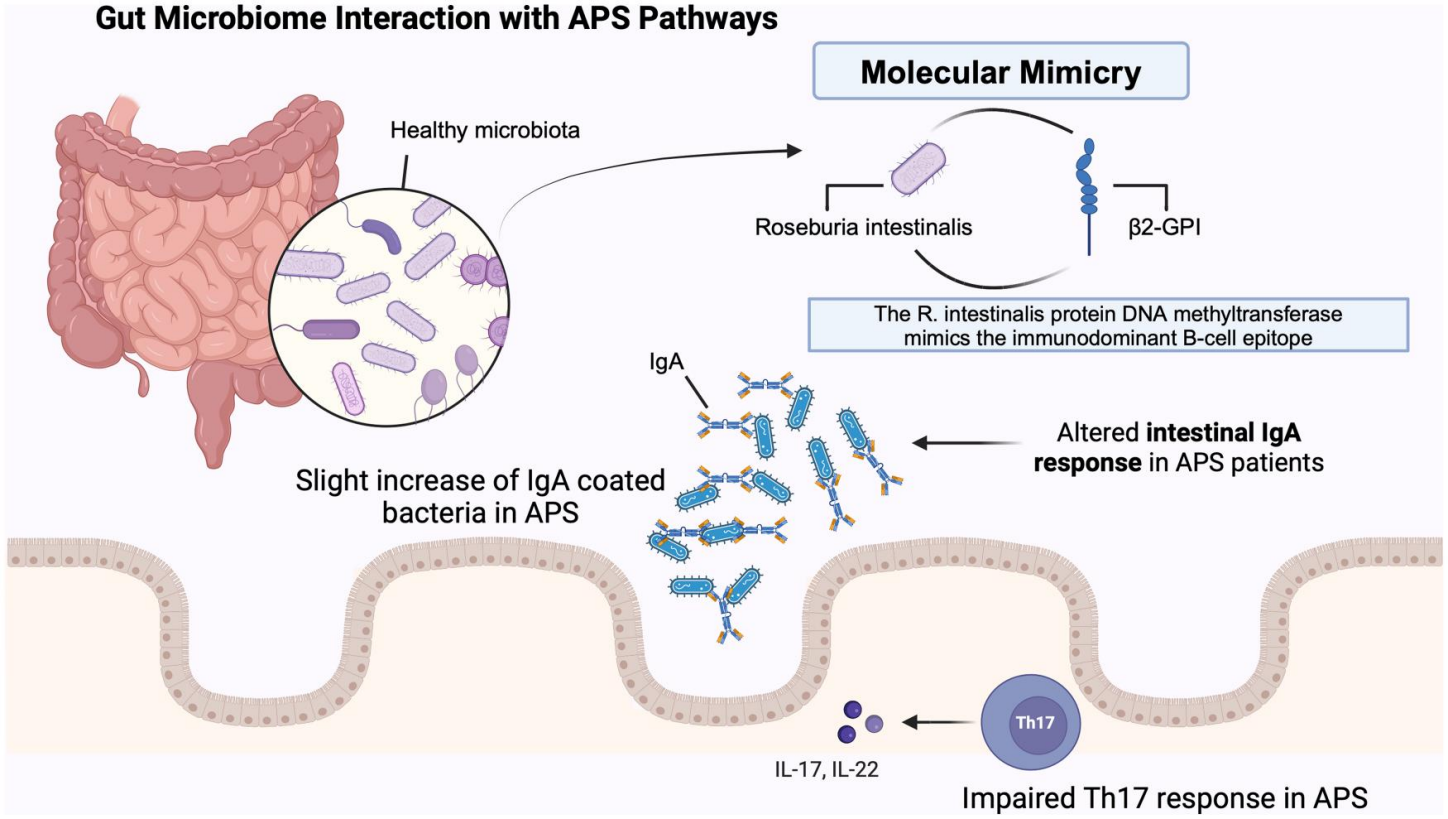
Triggers in APS. Potential interactions between the gut microbiome and APS pathways. It has been suggested that certain gut microbiota, such as *Roseburia intestinalis*, may mimic β2 glycoprotein I (β2-GPI), a key autoantigen in APS. This mimicry could trigger an autoimmune response, contributing to the production of anti-β2-GPI antibodies. An altered intestinal IgA response in APS patients is shown, with a slight increase in IgA-coated bacteria compared to healthy controls, indicating abnormal immune interactions with gut microbes. Additionally, an impaired Th17 response is depicted, marked by reduced IL-17 and IL-22 levels, which contribute to immune dysregulation in APS. Together, these components highlight the potential role of the gut microbiome in influencing immune responses and disease progression in APS

The relationship between the gut microbiome and autoimmune disorders, particularly APS, has garnered increasing attention due to its potential role in modulating immune responses [14, 33, 34]. Evidence suggests that gut microbiota may contribute to APS development through mechanisms such as molecular mimicry, where gut microbes express proteins homologous to APS autoantigens, leading to an autoimmune response (Fig. 2) [14]. It has been suggested that patients with APS often show an altered intestinal IgA response, indicating abnormal immune interactions with the microbiota. Although significant differences in microbial diversity between APS patients and healthy controls have not been widely observed in human studies, the correlation between a-β2GPI antibody titres and homologous microbial proteins implies a relevant interaction that warrants further exploration [14].

Environmental factors, including microbial and viral agents, play a role in APS by potentially inducing aPL through mechanisms like molecular mimicry, which can trigger autoantigen production and contribute to the onset of APS [15, 32]. While microbiota-targeting therapies are still experimental, murine studies suggest that modifying the gut microbiome could influence APS disease activity, offering a potential therapeutic avenue [14, 15, 66]. Research into the gut microbiome’s involvement in APS pathogenesis is promising, with future therapies potentially harnessing this relationship to modulate immune responses and reduce disease severity. However, more studies are needed to confirm these interactions and develop effective microbiota-based treatments for APS and other autoimmune diseases [14, 15].

#### APS in the era of COVID-19: a new paradigm

APS and COVID-19 share overlapping concerns regarding thrombotic complications [67, 68]. COVID-19 induces a hypercoagulable state, raising concerns for APS patients already at risk of thrombosis. Reports suggest that COVID-19 may transiently trigger aPL, but their role in thrombotic events remains unclear [68]. Although some studies report high aPL prevalence in COVID-19 patients, the antibodies do not consistently correlate with increased thrombosis, and the risk of developing persistent APS-like manifestations or chronic APS due to COVID-19 appears low [67–69]. Concerns about vaccine-induced aPL positivity, particularly with adenoviral vector vaccines (e.g. AstraZeneca), have emerged. Nevertheless, the risk remains low, and mRNA-based vaccines have shown minimal thrombotic complications in APS patients [68, 70]. No evidence supports that COVID-19 vaccines trigger APS in those without pre-existing aPL [68, 70].

During the pandemic, APS patients, particularly those hospitalized with COVID-19, required close monitoring due to increased thrombosis risk, with preventive anticoagulation often recommended. Encouragingly, studies suggest that even APS patients with triple aPL positivity tolerate COVID-19 vaccines well, alleviating vaccine hesitancy in this high-risk group [69]. Continued research will further clarify the relationship between aPL and COVID-19-related thrombosis.

#### Artificial intelligence in APS: predicting and managing thrombotic risks

Artificial intelligence (AI) is becoming increasingly valuable in predicting and managing thrombotic risks in APS [71, 72]. Machine learning models have been used for risk stratification, analysing complex data sets such as thrombin generation and thrombin dynamics, and achieving diagnostic accuracies as high as 92% in APS patients, even when they are on anticoagulation therapy [73]. This high accuracy suggests that AI can provide clinicians with a reliable tool for identifying at-risk patients without requiring therapy interruption.

AI-driven models assess various factors in personalized treatment plans, including LA, a-β2GPI and aCL antibodies. By combining clinical and laboratory data, AI models have improved the ability to predict thrombotic events and adjust treatment strategies accordingly, with models demonstrating a predictive accuracy of over 85% in some studies [72]. The future of AI in APS diagnosis and management holds great promise as these technologies evolve to incorporate real-time monitoring and dynamic treatment adjustments. With continuous input from biomarkers and imaging data, AI could further enhance risk prediction and therapy optimization, potentially improving outcomes for the estimated 30–50% of APS patients at the highest risk for recurrent thrombotic events [73].

#### Quality of life and long-term outcomes in APS

APS significantly affects patients’ quality of life (QoL), particularly mental and psychological health. Research shows reduced health-related QoL (HRQoL) in APS patients, especially those experiencing arterial thrombosis [74–77]. Zuily *et al.* reported that patients with APS had lower mental component scores (MCS: 40.6 ± 16.5) compared with the general population, particularly those with SLE-associated APS [77]. Depression, anxiety and mood disorders are prevalent, especially in patients with obstetric complications [74].

The SF-36 and EuroQol-5D are commonly used to assess HRQoL in APS, evaluating physical and mental health domains. APS-specific indices, such as DIAPS, are also being developed to capture disease-related damage that influences QoL [16, 25, 74]. Improving long-term QoL requires managing organ damage, reducing thrombotic event recurrence, and addressing neurocognitive impairments through optimized anticoagulation therapy and psychological support [74–76]. Routine HRQoL assessment is essential for tailoring interventions, and early use of immunosuppressive therapies may reduce long-term complications, thereby enhancing the overall QoL in APS patients [74, 77].

## Future directions and conclusions

In conclusion, advancements in APS management focus on improving diagnostic precision and developing targeted treatments, as shown by emerging studies and ongoing clinical trials (Table 1) [11, 78–84]. Risk stratification, particularly for patients with complex APS profiles, remains a significant challenge, but AI-driven models are being tested to predict thrombotic events using biomarkers and clinical data [11, 71, 73]. For clinicians, combining biomarkers (e.g. non-traditional antibodies, miRNAs) with advanced imaging techniques like MRI is crucial in personalizing treatment plans and improving early detection and management [11, 84]. Future studies and trials are expected to validate these approaches, leading to more personalized, effective treatments for APS.

**Table 1.** Ongoing clinical trials in APS: exploring emerging therapies and future directions

Study name	Sponsor	Phase	Intervention	Mechanism of action	Key focus	Completion date	References
Telitacicept in Primary APS Patients	Peking Union Medical College Hospital	Phase 2	Telitacicept	Fusion protein that neutralizes the activity of B-lymphocyte stimulator and a proliferation-inducing ligand.	APS with extra-criteria manifestations	31 December 2022	[78]
The BeLimumab Antiphospholipid Syndrome Trial (BLAST)	University of Turin, Italy	Phase 2/Phase 3	Belimumab	Blocks B cell activating factor.	Refractory APS	1 January 2025	[79]
Anti-CD38 Antibody Treating APS with Thrombocytopenia	Institute of Hematology & Blood Diseases Hospital, China	Phase 2	Anti-CD38 antibody	Blocks CD38 activity which includes antibody-dependent cellular cytotoxicity, antibody-dependent cellular phagocytosis, complement-dependent cytotoxicity, direct cellular apoptosis, and extracellular ectoenzyme activity modulation.	APS with secondary thrombocytopenia	August 2025	[80]
Safety and Efficacy of Zanubrutinib in the Treatment of APS	Zhang Lei, MD, Institute of Hematology & Blood Diseases Hospital	Phase 2	Zanubrutinib	Small-molecule kinase inhibitor that works by binding to a cysteine residue in the active site of Bruton tyrosine kinase.	APS with secondary thrombocytopenia	30 June 2024	[81]
Daratumumab in Primary Antiphospholipid Syndrome (DARE-APS)	National Institute of Allergy and Infectious Diseases (NIAID)	Phase 1/Phase 2	Daratumumab	Anti-CD38 (see mechanism above).	Safety of daratumumab in APS	29 October 2025	[82]
IMPACT Study: IMProve Pregnancy in APS With Certolizumab Therapy	University of Utah	Phase 2	Certolizumab pegol	Works by attaching to and neutralizing tumor necrosis factor-alpha.	Improving pregnancy outcomes in APS patients	December 2025	[83]
Phase 1b Trial of RAY121 in Immunological Diseases (RAINBOW Trial)	Chugai Pharmaceutical	Phase 1	RAY121	Strong inhibitor of classical complement pathway.	Safety, tolerability, pharmacokinetics, pharmacodynamics, immunogenicity and preliminary efficacy of RAY121 in immunological diseases (including APS).	30 June 2026	[84]

## Data Availability

No new data were generated or analysed in support of this article.
